# Guardians of the Gut: Enteric Defensins

**DOI:** 10.3389/fmicb.2017.00647

**Published:** 2017-04-19

**Authors:** Sumathi Sankaran-Walters, Ronald Hart, Chantelle Dills

**Affiliations:** Medical Microbiology and Immunology, University of California, DavisDavis, CA, USA

**Keywords:** enteric alpha defensins, mucosal immunity, paneth cells, IBD microbiota research, antimicrobial peptide

## Abstract

Enteric defensins likely play a key role in the management of the human microbiome throughout development. The functional and mechanistic diversity of defensins is much greater than was initially thought. Defensin expression and overall Paneth cell physiology likely plays a key role in the development of colitis and other inflammatory or dysbiotic diseases of the gut. As our understanding of enteric defensins grows, their potential as tools of clinical intervention becomes more apparent. In this review, we focus on the function and activity of Paneth Cell defensins and highlight their role in disease.

## Introduction

In general, the term “antimicrobial peptide” is used to describe a polypeptide chain of 5–100 residues that has broad-spectrum antimicrobial activity at one or more sites throughout the body (Bahar and Ren, 2013). While there are over 5,000 distinct antimicrobial peptides described, they fall into three major categories: cathelicidins, defensins, and histatins, each having various domains and functions (Bahar and Ren, 2013; Zhao et al., 2013). Defensins, specifically enteric defensins (noted as HD5 and HD6, or alpha defensin 5 and 6), are one type of these antimicrobial peptides. These peptides have experienced a relatively large resurgence in studies into their functions and potential because, until recently, it was difficult to isolate them in quantities sufficient to study. New techniques for the synthesis of these peptides, however, have opened the door to greater understanding of their functional importance (Wu et al., 2004).

With the rise of antimicrobial resistance, research into new antibiotic therapies is becoming increasingly important. With this in mind, much effort is being devoted to understanding the scope and functional importance of defensins, especially those involved in the maintenance of the gut epithelium as a barrier to enteric infection. As such, defensins and other natural peptide antibiotics may prove to be promising sources of novel therapies for enteric infection. Within this review, we will explore current understanding and hypotheses regarding the structure, regulation, and physiological function of enteric defensins, and close with a discussion of potential clinical interventions using defensins.

## Overview: α- and β-defensins, and their localization

In humans, there are 17 described defensins found at various sites of the body (Jarczak et al., 2013). These 17 fall into two major categories, α-defensins and β-defensins, with a third category, θ-defensins having recently been described in the **leukocytes** (see Glossary) of rhesus macaques but not yet observed in humans (Lehrer et al., 2012). Structurally, α- and β-defensins differ in the length of the peptide segments that separate six specific cysteine residues within the structure, and the way that these cysteines pair to each other via disulfide bonds (Ganz, 2003). Distributionally, defensins within both categories are secreted from a number of cells, including leukocytes, **neutrophils** (see Glossary) and several types of epithelial cells. For our purposes, it is important to note all known enteric defensins in humans are alpha defensins, specifically Human Defensin 5 (HD5) and Human Defensin 6 (HD6).

There are six α-defensins described in humans, designated as DEFA 1–6. DEFA 1–4 are also called Human Neutrophil Peptides 1–4 as they are most often expressed by neutrophils, commonly making up as much as 50% of the total protein content in these cells (Date et al., 1994; Faurschou and Borregaard, 2003). These peptides are thought to be involved in the cell-killing activity of cells of the innate immune system, and are key players in systemic innate immunity (Khine et al., 2006).

DEFA 5 and 6, also known as Human Defensin 5 and Human Defensin 6, are produced only by **Paneth cells** (see Glossary) of the small intestine (Lisitsyn et al., 2012). However, it is still unclear whether the expression of HD5 and HD6 is consistent along the length of the small intestine. Little is currently known about the local expression patterns of defensins along the human intestinal tract beyond the fact that they are expressed by Paneth cells, found only in the small intestine. It is thus possible that there may be differences in human enteric defensin composition along the length of the small intestine which may impact the local microbiota (Nakamura et al., 2016).

## Structure and mode of action

Defensins, while diverse in overall structure, are related through six invariant cysteine residues that form 3 intramolecular disulfide bridges found in all defensins (Xie et al., 2005). Additionally, there is a highly conserved salt bridge formed between Arg^6^–Glu^14^ that is thought to play an important role in the processing and stabilization of the final peptide (Rajabi et al., 2008). Until recently, the amphipathic nature of defensins was thought to be the most important factor in their cell-inhibiting ability. To explain, antimicrobial peptides are rich in both cationic and hydrophobic residues making them well-suited to interactions with and integration into cell membranes, which are also made of amphipathic phospholipids (Ganz, 2003). While the specific mechanism of HD5 and HD6 is currently an area of intense research, the neutrophil defensins (DEFA 1–4) are known to affect membrane permeabilization and cause the formation of excess potassium channels, leading to cell death (Lehrer et al., 1989). While interactions with cell membranes are a part of the action of the enteric defensins, new evidence suggests that their modes of action are more diverse than this.

Recent evidence has suggested that the main method of cell killing for HD5 may be similar to the neutrophil peptides, in that it interacts with and disrupts bacterial cell membranes. Studies using x-ray crystallography have determined that HD5 forms dimers using leucine residues and substitution of Leu^29^ with other amino acids greatly diminishes the cell-killing abilities of HD5 (Szyk et al., 2006; Rajabi et al., 2008, 2012). This suggests that dimerization is important to the function of HD5 and is also likely related to the hydrophobicity of the peptide (Szyk et al., 2006; Zhang et al., 2010; Rajabi et al., 2012; Zhao et al., 2012). Substitution of this leucine residue with unnatural amino acids of varying hydrophobicity showed that bactericidal activity of HD5 increased with side chain length of the substituted amino acid, further emphasizing a key role for hydrophobic interactions in the cell-killing mechanism of HD5 (Szyk et al., 2006; Chu et al., 2012; Rajabi et al., 2012; Mathew and Nagaraj, 2015). However, recent studies have also suggested that HD5 may exert its effects from within the cytoplasm of the target cell. Recent analysis has shown that *Escherichia coli* exposed to HD5 undergoes distinct morphological changes that include **bleb** morphology, cellular elongation, and clumping (Chileveru et al., 2015). GFP analysis showed that during these morphological events, oxidized HD5 accumulated within the bacteria at sites of cell division and the cell poles, suggesting that the observed morphological changes are related to intracellular HD5 (Chileveru et al., 2015). Taken together, this suggests that HD5 has multiple mechanisms through which it exhibits inhibitory function on bacteria.

Until recently, the importance of HD6 has not been well-understood. Previously, it has been shown that HD6 exhibits relatively low levels of bactericidal activity at biological concentrations (Ericksen et al., 2005). However, recent studies have identified a non-bactericidal role for HD6 in host defense. HD6 spontaneously self-assembles into multi-peptide nanonets upon contact with bacterial appendages such as flagella and fimbrae (Chu et al., 2012). This function gives HD6 a key role in aggregating and sequestering bacteria that enter the crypts of the small intestine; rather than direct cell killing, it appears that HD6 puts bacteria in arrest.

As with most defensins, it appears that environmental factors play a key role in the activity of HD6. As an example, in the presence of a reducing agent, HD6 becomes more hydrophobic and this reduced form inhibits the growth of *Bifidobacterium adolescentis* to a much greater degree than the unreduced form (Schroeder et al., 2015). This suggests that local and momentary conditions within the physiological environment may be able to direct the action of defensins in a dynamic fashion, such that they only exhibit bacterial killing under certain conditions. Recent evidence has suggested that this observation may not be restricted to HD6: it was also found that the effectiveness of HD1–4 as antimicrobials varies with the pH and reducing conditions of the environment (Schroeder et al., 2015). This suggests that defensins have certain conditions in which they function optimally; as such, environmental factors may play a role in the regulation of the activity of enteric defensins.

Because most defensins exert their effect on the membranes of cells, scientists are interested in why defensins are essentially harmless to host cells at concentrations that kill microbial cells (Wu et al., 2010; Lioi et al., 2012). One theory to explain this hinges upon compositional differences in the cell membranes of bacterial and mammalian cells. Because bacterial cell membranes contain large numbers of acidic phospholipids, such as phosphatidylglycerol and cardiolipin, their surfaces hold significantly more negative charge than membranes of eukaryotic cells (Wimley et al., 1994; Bevins, 2005; Matsuzaki, 2009). Antimicrobial peptides, and thus defensins, being cationic are more likely to interact with bacterial cell membranes than mammalian cell membranes because of this difference in surface charge (Llenado et al., 2009; Clevers and Bevins, 2013; McSorley and Bevins, 2013). Another hypothesis to explain the selective toxicity of antimicrobial peptides relies on differences in the membrane potential of microbes and mammalian cells. Microbes tend to have a significantly larger charge separation across their membranes than mammalian cells which may allow cationic defensins to selectively target microbes (Lichtenstein et al., 1988; Yeaman and Yount, 2003; Zhang et al., 2010; Vega and Caparon, 2012).

While much effort is devoted to understanding the function of defensins in relation to bacteria and other living organisms, defensins have also been shown to exhibit direct antiviral activity and may play a key role in protecting hosts from enteric viral infections (Wilson et al., 2016). As such, many examples of the antiviral properties have been uncovered in recent research. One example of this lies with JC polyomavirus, the pathogen responsible for progressive multifocal leukoencephalopathy. It was found that HD5 blocks infection with JC polyomavirus by stabilizing the viral capsid after infiltration of host cells, preventing release of the viral genome (Zins et al., 2014). This is only one example of HD5 exerting effects on a non-enveloped virus. Molecular modeling has also shown that HD5 can interact with the vertex region of the human adenovirus capsid, preventing uncoating and, thus, inhibiting infectivity (Flatt et al., 2013). However, mouse models have shown that *in vivo*, the antiviral effect of HD5 on adenovirus is distinct from that observed in culture; rather than interacting directly with the viral capsid, it was shown that an antiviral antibody response was mounted in response to mouse adenovirus, and that this response was blunted in mice deficient in alpha defensins, suggesting that HD5 may play a role in directing the activity of the host's adaptive immune system (Gounder et al., 2016). Thus, enteric defensins appear to be multifaceted in their effects on viral pathogens. Despite the fact that much of our knowledge of the antimicrobial activity of defensins hinges on their interaction with lipid membranes, these examples of HD5's interaction with non-enveloped viruses provide evidence for modes of biological inhibition beyond interactions with membranes, including through direct interaction with viral proteins and direction of host immunity (Wiens et al., 2014).

## Regulation, expression, and release

DEFA 5 and 6 are expressed and secreted primarily by Paneth cells, found at the base of crypts of Lieberkühn in the small intestine. While Paneth cells have a number of functions, one of their primary functions is the storage and release of these defensins (Bevins and Salzman, 2011). Our current understanding suggests that defensins are probably not produced for immediate release by Paneth cells, but are instead constitutively produced in an inactive form and stored in this form in secretory granules for release at a later time (Cunliffe et al., 2001; Ouellette, 2011).

Evidence suggests that trypsin found in Paneth cells converts pro-HD5 into the active form found in the gut lumen (Ghosh et al., 2002). A similar mechanism has been observed with murine alpha defensins, also known as **cryptdins** (see Glossary). CRS4C-1, a member of these mouse cryptdins, is stored in Paneth cells and secreted from them as non-microbicidal pro-CRS4C-1, which is cleaved into its active form in the intestinal lumen by matrix metalloproteinase-7 (Shanahan et al., 2010; Andersson et al., 2012). While understudied, further investigations into MMP regulation and expression may reveal an MMP-related mechanism for the indirect regulation of broad-scale defensin activity (Yan and Boyd, 2007). Interestingly, studies have shown that Human Neutrophil Peptide 1 can affect the expression of various MMPs under certain conditions, suggesting that the relationship between defensin activation and metalloproteinase activity may not simply be one-way (Musrati et al., 2016).

In general, there are a large number of stimuli responsible for triggering the degranulation of Paneth cells, and thus release of defensins into the intestinal lumen. Various components of the bacterial cell wall have been shown to trigger Paneth cell degranulation. Among these components are LPS, lipotechoic acid, lipid A, and muramyl dipeptide (Ayabe et al., 2000) all of which stimulate TLRs. This suggests that defensin release is associated with TLR activation. Further, mice treated with CpG-oligodeoxynucleotide undergo rapid Paneth cell degranulation (Rumio et al., 2012). As an agonist of TLR9, this further implicates TLRs in the defensin response. Defensin secretion in response to bacteria and bacterial antigens appears to happen in a dose-dependent fashion (Ayabe et al., 2000). Further studies suggest that Paneth cells directly sense commensal bacteria of the gut through a pathway mediated by the MyD88 toll-like receptor (Vaishnava et al., 2008).

Defensin release can also be triggered by signaling molecules not directly related to abnormalities in the microbes of the gut. For example, Paneth cells exposed to pro-inflammatory cytokine IFN-γ undergo immediate and complete degranulation (Farin et al., 2014). It is hypothesized that *in vivo*, this IFN-γ is secreted by iNKT cells within the gut epithelium, but this remains a question for further study. Nonetheless, this suggests that defensin release may also be triggered by general disruption of the gut epithelium, such as during inflammation. This hypothesis is further supported by evidence that exposure of Paneth cells to interleukin 13, a cytokine also involved in the inflammatory response, induces Paneth cell degranulation (Stockinger et al., 2014). This suggests that defensins are released in response to a wide variety of stimuli, including gut dysbiosis and damage to the epithelium itself.

## Host microbiome and physiological effects

The gut mucosa, and associated factors of the immune system, function as a first line of defense against opportunistic pathogens of the gut by preventing invasion and colonization of tissue by microbes that could potentially damage the host. The enteric defensins, HD5 and HD6, are thought to be key contributors to innate immunity in the gut and are thus intricately linked to the health of the gut mucosa and the host as a whole. Therefore, it is important to examine the large-scale effects of defensins on the host's microbiome and overall gut physiology.

New evidence is beginning to uncover the effects of defensins on host microbiomes. For example, mice transgenic for DEFA5 have been shown to have a lower proportion of gram-negative Bacteriodetes in their gut, compared to wild-type mice unable to express HD5 (Salzman et al., 2010). Consequently, the absence of active HD5 was correlated with an increase in the proportion of Firmicutes over Bacteriodetes (Salzman, 2010; Salzman et al., 2010). Further, recent studies have suggested that both systemic and ileal defensins are either preserved or increased in expression with age (Castañeda-Delgado et al., 2013; Cunha et al., 2014). As a result, defensins may play a mechanistic role in the microbiota changes that can be observed with aging (Mariat et al., 2009; Salzman, 2010; Jandhyala et al., 2015).

Paneth cell-associated pathology can show the role of defensins in host immunity. As an example, graft-vs.-host disease (GVHD) is a condition following stem cell or bone marrow transplants in which the donor cells attack tissues of the recipient's body (Ferrara et al., 2009). The Paneth cells of GVHD-afflicted mice are damaged, resulting in a significant decrease in the expression of α-defensins. GVHD-affected mice saw a dramatic expansion of *E. coli* communities in the gut and eventual **septicemia** (see Glossary), suggesting Paneth cell defensins play a role in the suppression of pathogenic communities within the gut (Eriguchi et al., 2012). While speculative, this function may not be a result of only direct killing of pathogens by defensins, but also by their promotion and cultivation of “healthy” microbial communities that outcompete pathogens. Further evidence for the protective effects of defensins can be seen in mice transgenic for DEFA 5. Mice with the human defensin gene were shown to be resistant to oral challenge with pathogenic *Salmonella typhimurium*, suggesting a direct role for Paneth cell defensins in host protection against pathogenic insult (Salzman et al., 2003).

More broad-scale diseases of the gut can also serve to highlight potentially interesting features of Paneth cell defensins and the relationship between innate immunity and normal gut physiology. It is known that bowel dysfunction, especially **ileitis** (see Glossary), is associated with abnormalities in gut microbiota; further, Paneth cell dysfunction is known to be associated with irritable bowel disease and other inflammatory conditions of the gut (Tamboli et al., 2004; Shi, 2007; Simmonds et al., 2014). However, it is unclear whether defensin-related dysfunction (and associated Paneth cell function) appears secondary to intestinal **dysbiosis** (see Glossary), or if Paneth cell abnormalities lead to changes in intestinal flora and associated intestinal inflammation. Evidence for microbe-driven ileitis comes from observations that transfer of dysbiotic microbial communities to germ-free mice induces symptoms similar to Crohn's disease, with loss of Paneth cell secretion of defensins following these inflammatory conditions (Schaubeck et al., 2016). However, a growing set of observations suggests a series of events that are contrary to this. Multiple Paneth cell-specific genetic variants have been identified in mice affected by ileitis. In mice, Autophagy Related 16 like 1 (ATG16L1) deficiency is known to be associated with Paneth cell dysfunction, specifically having an effect on granule exocytosis; thus, the absence of ATG16L1 results in abnormalities in the release of enteric defensins into the gut lumen (Cadwell et al., 2008). Further, ATG16L1 deficiency, when found in conjunction with Paneth cell-specific deletion of the gene that codes for **X-box binding protein-1** (Xbp1), results in spontaneous ileitis (Adolph et al., 2013). These two observations suggest that dysfunction in the release of defensins into the gut lumen may bring about ileitis. In reality, the relationship between Paneth cell function and dysbiotic inflammation is likely more complex than either of these explanations alone.

Previous evidence has suggested that normal Paneth cell function is related to zinc intake by individuals. Until recently, the extent of our understanding was that zinc deficiency is associated with a significant reduction in the number of storage vesicles (granules) within the Paneth cells of individuals (Kelly et al., 2004). However, more recent research has explored the mechanism by which zinc intake and Paneth cell function may be related. Reduced HD5 has been shown to be a potent zinc-ion chelator. Further, reduced HD5 bound to zinc is resistant to proteolytic breakdown, where non-chelated HD5 is quickly broken down by proteases such as trypsin (Zhang et al., 2013). As such, dietary zinc seems necessary to the persistence of reduced HD5 within Paneth cells. Until recently, the function of reduced HD5 in the gut was unknown despite its consistent presence *in vivo*. New evidence suggests that reduced HD5 may act to sequester and neutralize free bacterial LPS in the gut, blunting the associated inflammatory response (Wang et al., 2016).

Paneth cell associated defensins have also gained prominence in systemic diseases such as metabolic syndrome. Mice that were fed high fat diet and were deficient in Vitamin D had decreased expression of Paneth cell-specific alpha-defensins including α-defensin 5 (DEFA5), MMP7 a defensing activator, as well as tight junction genes in the ileum. As previously shown this combination allows for increased gut permeability and microbial translocation, dysbiosis, endotoxemia, and systemic inflammation which underlie metabolic syndrome (Su et al., 2016). Conditions such as cardiovascular disease, cerebrovascular insufficiency, and others are tied to systemic inflammation as well (Benakis et al., 2016; Singh et al., 2016). Unanswered questions include the role of Paneth cell Defensins in such systemic conditions.

## Clinical applications

Due to their key role in the innate immune system, defensins show promise in many clinical applications.

Tumor Necrosis Factor is a pro-inflammatory cytokine found in increased concentrations in the gut mucosa of patients with active colitis (Olsen et al., 2007). As such, anti-TNF therapy has been employed in the treatment of colitis and other inflammatory diseases of the bowel with varying degrees of success (Danese et al., 2013). Recent evidence, however, shows that mucosal expression patterns of certain defensins can be used to predict the potential efficacy of anti-TNF treatment. More specifically, it was found that a positive response to anti-TNF therapy was associated with increased mucosal expression of HD5 (Magnusson et al., 2016).

Defensins also show promise as sources of new antibiotics. HD5 has been artificially modified to enhance its antibacterial properties. It was found that substitution of Glu21 with Arg, and Thr7 with Arg greatly increased the antibacterial activity of HD5 despite hindering dimerization among individual peptides (Wang et al., 2015).

HD5 may be implicated in the repair of epithelial tissues following damage. Treatment of murine wound beds with HD5 stimulates the migration of LGR stem cells into the wound bed, promoting epithelial repair and wound healing (Lough et al., 2013). The effect of HD5 on LGR-marked stem cells may be important in the repair of the gut epithelium following damage. Further, HD5 has been shown to up-regulate several key mRNA transcripts in the Wnt signaling pathway (Lough et al., 2013). Recent observations have shown TCF-1 mediated Wnt signaling to be disrupted in Crohn's disease (Beisner et al., 2014). These two observations may provide the basis for questions into restorative effects of HD5 in Crohn's disease through upregulation of Wnt signaling transcripts. However, the study of defensins is open with many important questions the answers to which could have valuable clinical applications (Table 1).

**Table 1:** **Some unanswered questions in the study of Paneth Cell enteric defensins**.

1.	Do Enteric Defensins have a role in innate immune responses to a broad spectrum of viral pathogens?
	Within this review, we've discussed multiple avenues through which HD5 can affect specific non-enveloped viruses. It remains to be seen if enteric defensins have a non-specific, and generalized response to viral pathogens in the gut.
2.	Is there a recognition pattern like PRR's that can directly activate defensins?
	A wide variety of TLR agonists and MAMPs have been shown to be associated with the release of enteric defensins. Further, TLR9-specific agonists have been associated with Paneth cell degranulation, implicating TLR9 in the defensin response. However, in general, the variety of pathways through which defensin release is triggered is not well understood.
3.	What is the role of defensins in the activation and resolution of inflammation?
	Pro-inflammatory cytokines have been implicated in paneth cell extrusion, but it is unclear if abnormalities in defensin release are implicated in the initiation of inflammation, through dysbiosis or otherwise. What role might defensins play in restoring healthy gut communities as a means of ameliorating intestinal inflammation?
4.	How is overall host nutrition related to defensin function and release?
	Proper amounts of zinc, vitamins etc. are necessary to certain functions of HD5 what other micronutrients may be essential to the function of enteric defensins? What role may this play in host health and immunity?

## Concluding remarks

Recently developed advances in the field of antimicrobial peptides have fueled new interest into properties of the human enteric defensins, HD5, and HD6 (Stange, 2017). Novel discoveries have been made with regard to the structure, functional diversity, and effect on host microbiota of these peptides (Sugi et al., 2017; Zhang, 2017). Many of these discoveries point to a much greater role for defensins in host health and immunity than previously thought. The potential for enteric defensins as tools of clinical intervention grows as our understanding of their role in host health and immunity increases.

## Author contributions

RH and CD researched and reviewed in order to create the manuscript. SS oversaw and finalized the manuscript.

## Funding

The authors of this review were funded by the NIH: NHLBI on award 1R21HL126562-01A1.

### Conflict of interest statement

The authors declare that the research was conducted in the absence of any commercial or financial relationships that could be construed as a potential conflict of interest.

## Glossary

*Leukocyte*: A broad class of immune cells found circulating in the blood. Comprised of basophils, eosinophils, basophils, monocytes, and lymphocytes.
*Neutrophil*: An immune cell found in the blood that neutralizes microorganisms through phagocytosis and the release of antimicrobial proteins. These are the most abundant type of leukocyte.
*Paneth cell*: Cell found in the gut epithelium that is responsible for producing and secreting enteric defensins in mammals. Found at the base of crypts in the small intestine.
*Bleb*: A protrusion of the plasma membrane of a cell. The presence of bleb morphology suggests structural dysfunction within the cell it is observed in.
*Cryptidins*: Antimicrobial peptides found in the gut of mice. These are considered by many to be the murine equivalent of defensins.
*Septicemia*: The presence of bacteria in the blood.
*Ileitis*: An inflammatory condition of a portion of the small intestine known as the ileum.
*Dysbiosis*: An abnormality in the microbial community of a host
